# Adjunct Diagnostic Value of Transcranial Magnetic Stimulation in Mucopolysaccharidosis-Related Cervical Myelopathy: A Pilot Study

**DOI:** 10.3390/brainsci9080200

**Published:** 2019-08-14

**Authors:** Mariagiovanna Cantone, Giuseppe Lanza, Alice Le Pira, Rita Barone, Giovanni Pennisi, Rita Bella, Manuela Pennisi, Agata Fiumara

**Affiliations:** 1 Department of Neurology, Sant’Elia Hospital, ASP Caltanissetta, Via Luigi Russo 6, 93100 Caltanissetta, Italy; 2 Department of Surgery and Medical-Surgical Specialties, University of Catania, Via Santa Sofia 78, 95125 Catania, Italy; 3 Department of Neurology IC, Oasi Research Institute—IRCCS, Via Conte Ruggero 73, 94018 Troina, Italy; 4 Referral Center for Inherited Metabolic Diseases, Department of Clinical and Experimental Medicine, University of Catania. Via Santa Sofia 78, 95125 Catania, Italy; 5 Child Neurology and Psychiatry, Department of Clinical and Experimental Medicine, University of Catania, Via Santa Sofia 78, 95125 Catania, Italy; 6 Department of Medical and Surgical Sciences and Advanced Technologies, Section of Neurosciences, University of Catania, Via Santa Sofia 78, 95125 Catania, Italy; 7 Department of Biological and Biotechnological Sciences, University of Catania, Via Santa Sofia 78, 95125 Catania, Italy

**Keywords:** motor evoked potentials lysosomal disorders, cortical-spinal tract, spinal cord compression, cervical myelopathy, clinical neurophysiology

## Abstract

Background: Cervical myelopathy (CM) is a common cause of morbidity and disability in patients with mucopolysaccharidosis (MPS) and, therefore, early detection is crucial for the best surgical intervention and follow-up. Transcranial magnetic stimulation (TMS) non-invasively evaluates the conduction through the cortico-spinal tract, also allowing preclinical diagnosis and monitoring. Methods: Motor evoked potentials (MEPs) to TMS were recorded in a group of eight patients with MPS-related CM. Responses were obtained during mild tonic muscular activation by means of a circular coil held on the “hot spot” of the first dorsal interosseous and tibialis anterior muscles, bilaterally. The motor latency by cervical or lumbar magnetic stimulation was subtracted from the MEP cortical latency to obtain the central motor conduction time. The MEP amplitude from peak to peak to cortical stimulation and the interside difference of each measure were also calculated. Results: TMS revealed abnormal findings from both upper and lower limbs compatible with axonal damage and demyelination in six of them. Notably, a subclinical cervical spinal disease was detected before the occurrence of an overt CM in two patients, whereas TMS signs compatible with a CM of variable degree persisted despite surgery in all treated subjects. Conclusions: TMS can be viewed as an adjunct diagnostic test pending further rigorous investigations.

## 1. Introduction

### 1.1. Mucopolysaccharidosis: A Brief Overview

Mucopolysaccharidosis (MPS) encompasses a group of inherited rare lysosomal diseases due to defective catabolism and storage of glycosaminoglycans (GAG) in the skeleton and soft tissues. MPS shows a wide and heterogeneous spectrum of clinical manifestations and severity, ranging from severe to very mild phenotypes that may be recognized only in adulthood. Diagnosis is based on the demonstration of elevated urinary excretion of mucopolysaccharides, enzyme deficiency, and genetic testing [1]. Enzyme replacement therapy (ERT) is available for MPS I, II, IVA, VI, and VII.

MPS I (Hurler or Scheie syndrome) and type II (Hunter syndrome) are the most common MPS types. The typical presentation is a pre-school age patient with developmental delay, short stature, recurrent ear and respiratory infections, and hepato-splenomegaly. Over time, the child develops hearing loss, cardiac valve disease, airway obstruction, skeletal contractures, distinctive facial appearance (macrocephaly, thick eyebrows, gingival hypertrophy, macroglossia, thickening of the lips and nasal alae), psycho-motor regression, and intellectual disability [1,2]. The characteristic radiographic findings, collectively termed dysostosis multiplex, include J-shaped sella turcica, oar-shaped ribs, pointing of the proximal metacarpals and metatarsals, and poorly developed acetabulum [3]. Untreated, the life expectancy is the second or third decade. Milder forms of MPS I and II have less prominent somatic findings and no CNS disease or intellectual disability [4].

MPS III is characterized by the predominance of neuropsychiatric rather than somatic problems, with developmental delay and intellectual disability manifesting early on [2]. In severe cases, development plateaus between 36 and 40 months, and these patients never learn to speak more than a few single words [5,6]. As the disease progresses, significant cognitive-behavioral (aggression, hyperactivity, decreased attention span, anxiety, and destructive behaviors) and sleep disturbances (difficulty falling asleep and frequent nighttime wakening) occur [5]. The progressive GAG deposition in the brain leads to seizures, spasticity, and feeding difficulties, gradually causing loss of language and ambulation till a vegetative state [4].

MPS IV (Morquio syndrome) includes type A and B, which are difficult to distinguish clinically, although the former is far more common. The age of onset is generally in the first few years of life [1]. Clinical manifestations are: short stature, pectus carinatum, forearm deformity, genu valgum, scoliosis, hip dysplasia, odontoid hypoplasia with atlanto-axial instability, and dental abnormalities. Non-skeletal features include corneal clouding, hearing loss, obstructive or restrictive lung disease, and cardiac dysfunction, whereas the intellect is normal [4]. A considerable spectrum of severity exists, and mildly affected individuals may have normal height and few clinical complications [7].

MPS VI (Maroteaux-Lamy syndrome) is characterized by storage of dermatan sulphate, but heparan sulphate metabolism is not impaired and, therefore, the intellect is generally normal [1]. This type shares many of the features of MPS I and II, including short stature, coarse facies, corneal clouding, airway obstruction, cardiac valve abnormalities, visceromegaly, inguinal and umbilical hernias, dysostosis multiplex, joint contractures, carpal tunnel syndrome, and hip dysplasia. The age of onset and disease progression are variable [4].

MPS VII is an ultra-rare disease. Notably, 40–45% of patients present with non-immune fetal hydrops [8,9], which is considered a relatively suggestive finding for MPS VII. In patients who survive beyond the neonatal period, common clinical features include intellectual disability, coarse facies, dysostosis multiplex, joint contractures, hepato-splenomegaly, scoliosis, corneal clouding, obstructive airway disease, cardiac valve disease, and cardiomyopathy [8].

### 1.2. Cervical Myelopathy in MPS

Cervical myelopathy (CM) is most frequently observed in MPS I, II, IV, and VI. It is the main cause of neurological morbidity and disability in these patients [10], with a significantly negative impact on their course of disease and quality of life [11]. CM is due to vertebral canal narrowing, which often results from the thickening of the dura mater and ligaments after GAG accumulation and fibrosis, epidural lipomatosis, and vertebral soma degeneration. Atlanto-axial subluxation due to odontoid hypoplasia may contribute to spinal cord compression and related clinical manifestations.

Although the effectiveness of ERT has been proven on different systemic complications of MPS, thus improving the lifespan of these patients, it does not have any effect on CM [12]. Given that an early detection of CM is associated with the best surgical outcome and post-operative course, both an accurate diagnosis and strict monitoring are recommended [13].

Magnetic resonance imaging (MRI) and computed tomography are the methods of choice to display spinal cord compression and vertebral abnormalities, respectively, although they do not provide any information on the functional status. In this context, motor evoked potentials (MEPs) to Transcranial Magnetic Stimulation (TMS) are diffusely used in clinical practice for the in vivo and real time non-invasive estimation of the excitation state and conduction velocity of the cortico-spinal tract [14], also allowing a preclinical diagnosis and monitoring [15,16,17,18,19,20,21,22]. In particular, MEP latency and central motor conduction time (CMCT) are viewed as reliable measures of the cortical-spinal myelination, whereas the MEP amplitude is known to reflect the status of excitation of the neuronal axons from the motor cortical areas to the spinal motoneurons till the muscles [14].

While several and robust TMS evidences are available in patients with different neurological disorders affecting the central motor system, to date few neurophysiological studies have been carried out in MPS-related CM [23,24,25,26,27]. Here, we applied TMS in patients with MPS to detect any electrophysiological sign, even at a subclinical level, of CM.

## 2. Materials and Methods

### 2.1. Subjects and Assessment

Eight patients (two males; median age 14.5 years, range 13.0–41.0) with a clinical, biochemical, and genetic diagnosis of MPS [28] were consecutively recruited from the “Referral Center for Inherited Metabolic Diseases, Department of Clinical and Experimental Medicine” of the University of Catania, Italy. Among these subjects, six (patient 1–6) had MPS IVA, whereas the remaining two (patient 7 and 8) had MPS VI.

As shown in Table 1, six subjects (1–4, 7, and 8) had previously received surgical decompression due to clinical and MRI evidence of CM, although four of them (1, 3, 7, and 8) still complained of neurological deficits. Regardless of previous surgery, at the time of the study, four patients with MPS IVA (2, 4, 5, and 6) did not have radiological evidences of CM. Finally, the two subjects with MPS VI had been treated with ERT for three years. The cervical cord compression and the CM at MRI were diagnosed by a trained neuroradiologist expert in vertebral and spinal cord diseases.

This research was performed according to the Declaration of Helsinki and all participants (or parents) gave their informed consent for inclusion before they participated in the study. This investigation was part of a larger multi-center study on clinical and molecular characterization of patients with MPS. The Ethics Committee of the “Azienda Ospedaliero-Universitaria Policlinico—Vittorio Emanuele” of Catania, Italy (PRIN 2012 code 20122EK9SZ_005) approved the study.

### 2.2. Transcranial Magnetic Stimulation

MEPs to TMS is included within the conventional diagnostic work-up of patients with suspected or overt CM, as well as in the peri- and post-operative course [14].

For diagnostic TMS, a high-power MagStim 220 mono-pulse stimulator (The Magstim Co., Ltd., Whitland, Dyfed, UK) connected to a 90 mm circular coil with an inner diameter of 5 cm, was employed to generate the motor responses. In a conventional exam, the patient is seated or lying on an armchair while recordings from distal limb muscles are performed. Standard EMG silver/silver chloride cup surface electrodes (9 mm diameter), jelly filled and applied over the first dorsal interosseous (FDI) and tibialis anterior (TA) muscles in a conventional belly tendon montage, were used for MEPs recordings from the contralateral side of stimulation [14].

The handle of the coil was pointing backward, whereas the coil center was positioned tangentially over Cz (international EEG 10–20 system) for MEP recordings from the FDI muscle and over Fz for MEP recordings from the TA muscle. A MEP to TMS in the relaxed muscle was first recorded. After that, MEPs with higher amplitude and shorter latency compared to the first response were obtained while patients produced a transient tonic muscular activity just enough to overcome gravity (approximately 10–20% of the maximal muscular activity). According to international guidelines, the shortest MEP latency was used for CMCT estimation. Similarly, only the MEP with the largest peak-to-peak size was considered for the analysis, given that a routine TMS exam evaluates the transcranially-induced motor response with the biggest amplitude. To determine the peripheral motor conduction time (PMCT), a motor nerve root stimulation was performed in all participants. The coil center was applied on the 7th cervical and 4th lumbar spinous process for upper and lower limbs, respectively. The time of conduction from the neurons within the motor cortex to those within the anterior horn of the spinal cord defines the CMCT, thus reflecting the central motor conductivity from the upper to the lower motoneuron. The PMCT by cervical or lumbar magnetic stimulation was subtracted from the MEP cortical latency to obtain the CMCT [14].

Given that the stimulation threshold for a 2.0 T magnetic stimulator (as that used in this study) is about 50–65% of the maximal output [29,30,31], motor responses were all obtained at 80% of the maximum stimulator output. The amplification and filtering (bandwidth 3–3000 Hz) of the motor responses were carried out by using a 2-channel Medelec Synergy system (Oxford Instruments Medical, Inc., Surrey, UK). As reference MEP values, we referred to those used in our TMS Lab, which were obtained from a large cohort of clinically and neuroradiologically intact subjects divided by age, height, and sex [32].

## 3. Results

**Figure 1:**
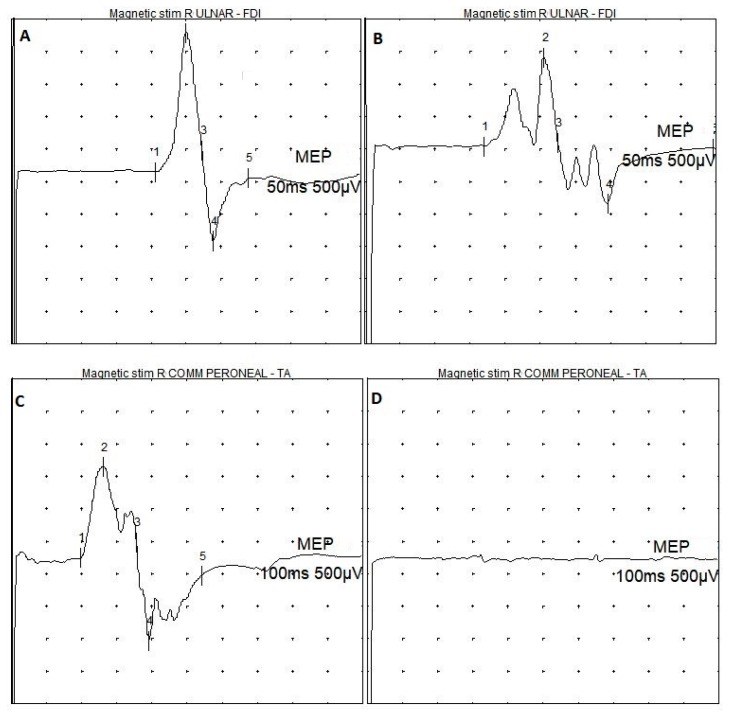
Examples of MEPs recordings in MPS patients. (**A**) normal MEP from the upper limb; (**B**) MEP with reduced amplitude and polyphasic shape from the upper limb; (**C**) normal MEP from the lower limb; (**D**) absence of transcranially-induced motor response from the lower limb. MPS = mucopolysaccharidosis; Magnetic stim = transcranial magnetic stimulation; R ULNAR = right ulnar nerve; R COMM PERONEAL = right common peroneal nerve; FDI = first dorsal interosseous muscle; TA = tibialis anterior muscle; MEP = motor evoked potential; 50 ms = temporal resolution of the screen (sweep) for upper limb recordings; 100 ms = temporal resolution of the screen (sweep) for lower limb recordings; 500 µV = amplification factor of the screen.

Table 2 summarizes the patients’ neurophysiological features. Overall, TMS was well tolerated and none complained of significant side-effects or discomfort during or after the examination. Figure 1 shows examples of both normal and pathological MEP recordings.

Among those who had underwent surgery for CM (patient 1–4, 7, and 8), MEPs were bilaterally absent from FDI and TA muscles in both patients with MPS VI (7 and 8), who already had a neurological impairment before treatment. In the treated patients with MPS IVA (1–4), MEPs were bilaterally absent from TA muscle in patient 3 as well as from the left TA muscle of patient 1. In the same patients (1–4), MEPs also showed reduced amplitude and polyphasic shape. Overall, CMCT was increased in three of the treated subjects from upper limbs (1, 2, and 4), whereas responses to cervical root stimulation could not be evoked in the other three (3, 7, and 8).

Regarding the four subjects without overt neurological symptoms (2, 4, 5, and 6), MEPs were abnormal in terms of reduced amplitude, increased latency, or polyphasic shape in at least one of the examined muscles in two of them (patient 2 and 4), whereas they were entirely normal in the other two (patient 5 and 6).

CMCT could not be bilaterally assessed at four limbs in three patients (3, 7, and 8) due to the absence of the evoked response by cervical or lumbar nerve root magnetic stimulation. Finally, no significant right-to-left difference was found for any of the TMS variables considered.

## 4. Discussion

In the present study, we found abnormal TMS findings from upper and/or lower limbs in six out of eight MPS patients, consistent with both diffuse axonal damage and demyelination. This suggests that a cervical spinal disease was clinically present before the occurrence of an overt CM and persisted, with a different clinical and neurophysiological level of severity, despite surgery. In this context, it should be acknowledged that patients with MPS may suffer from a wide spectrum of neurological symptoms that involves both CNS and the peripheral nerves and the musculo-skeletal system. In particular, they usually need neurosurgical intervention for CM or vertebro-spinal anomalies, although a spinal cord compression may occur and progress even in the absence of overt neurological symptoms [33].

Notably, MEPs response were bilaterally absent at four limbs from the two patients with MPS VI. This finding is compatible with a severe CM-related involvement of the cortico-spinal tract and suggests that this MPS type is particularly associated with an early-onset CM and related complications. Accordingly, recent recommendations from the “MPS VI Clinical Surveillance Program” conclude that patients with MPS VI, even from an early age, are all at high risk to develop CM and that, from the time of the diagnosis, monitoring with MRI should be performed [34]. Moreover, management of peri-operative course of MPS VI patients is often challenging and, therefore, the electrophysiological studies play a significant role in providing both surgical indication and proper timing, as well as in the monitoring of post-operative course. MEPs analysis also revealed a functional impairment even in two patients without a clear evidence of CM, thus allowing a preclinical diagnosis [23]. Therefore, TMS can be viewed as an extension of clinical examination and the functional counterpart of the neuroimaging techniques in assessing spinal cord disease, including the very early stages.

To date, the role of electrophysiological studies in detecting compressive myelopathy in patients with MPS has been investigated by few previous reports [23,24,25], and one study only has used TMS for the evaluation of the post-operative follow-up in a single patient with MPS VI [23]. In this frame, the intraoperative neurophysiological monitoring by using MEPs and somatosensory evoked potentials seems to be of pivotal interest as it provides relevant functional information during surgical procedures [27]. Since cervical cord compression in MPS is progressive and may produce rapid loss of sensory-motor functions in these patients (especially in those with type VI), surgery is indicated as soon as myelopathy is detected, even subclinically, as severely myelopathic subjects show little or no recovery after the operation [24,35], also at the TMS level, as confirmed by the present investigation.

It is worthwhile to mention that histological examination in a mice model of MPS type I showed a storage of GAG in the cortex and cerebellum, along with the evidence of a progressive inflammatory response that can contribute to the neurological deficit [36]. Based on its intrinsic properties, TMS might be considered as an additional tool able to disclose subclinical CNS involvement related to a neuroinflammatory status in MPS, a finding which has also been demonstrated in other autoimmune and metabolic disorders [37,38,39]. In this view, innovative neuromodulatory protocols based on non-invasive brain stimulation techniques might be applied to transiently modulate cortical excitability, synaptic plasticity, and functional connectivity [40,41,42,43,44].

The main limitation of this study is the small sample size, although MPS is a rare disorder. Nevertheless, these findings have to be interpreted with caution, since the small and heterogeneous data set does not allow to draw definite conclusions. Another caveat is that only patients with severe MPS VI phenotype were included, thus we cannot compare these findings with those from patients with mild phenotype. Finally, long term MEP data, before and after surgical intervention, are needed to confirm the role of TMS for prognostication purposes. Therefore, the results provided here should be considered descriptive and to be used as an adjunct test pending further rigorous investigations. Future studies and longitudinal exams are needed for early diagnosis, accurate prognosis, and adequate monitoring.

## Figures and Tables

**Table 1:** Clinical and demographic characteristics of MPS patients at the time of the study.

Patient	1	2	3	4	5	6	7	8
MPS type	IVA	IVA	IVA	IVA	IVA	IVA	VI	VI
Sex/age (years)	F/14	M/15	F/16	M/13	F/20	F/40	F/13	F/14
ERT (age, years)	-	-	-	-	-	-	+ (9)	+ (10)
Height (cm)	98	100	102	110	150	113	120	110
Spinal cord surgery (age, years)	+ (5)	+ (4)	+ (8)	+ (10)	-	-	+ (10)	+ (11)
Diffuse brisk tendon reflex	+	-	+	+	-	-	+	+
Limbs paresis/weakness	+	-	+	-	-	-	+	+
Walking assistance	+	-	+	-	-	-	-	+
MRI cervical cord compression	+	+	+	+	-	-	+	+
MRI cervical myelopathy	+	-	+	-	-	-	-	+

MPS = mucopolysaccharidosis; F = female; M = male; ERT = enzyme replacement therapy; MRI = magnetic resonance imaging; + = present; - = absent.

**Table 2:** Motor evoked potentials of MPS patients.

*N*	First Dorsal Interosseous Muscle											Tibialis Anterior Muscle								
	MEPs Amp (mV)		ID	Poly-Phasic Shape		MEPs Latency (ms)		ID	CMCT (ms)		ID	MEPs Amp (mV)		ID	MEPs Latency (ms)		ID	CMCT (ms)		ID
	R	L				R	L		R	L		R	L		R	L		R	L	
	*>2.8*	*>2.8*	*<4.0*	-	-	*<22.5*	*<22.5*	*<1.5*	*<7.6*	*<7.6*	*<1.5*	*>1.9*	*>1.9*	*<4.0*	*<31.2*	*<31.2*	*<4.1*	*<17.2*	*<17.2*	*<3.0*
1	**0.2**	**0.1**	0.1	+	+	19.2	20.0	0.8	**nr**	**10.1**	/	**0.3**	**nr**	/	29.3	**nr**	/	**21.2**	**nr**	/
2	**2.6**	**2.2**	0.4	-	+	16.5	17.1	0.6	**8.1**	**8.1**	0.0	3.2	**1.7**	1.5	19.6	21.5	1.9	12.3	14.0	1.7
3	**0.3**	**0.2**	0.1	+	+	19.4	20.6	1.2	**nr**	**nr**	/	**nr**	**nr**	/	**nr**	**nr**	/	**nr**	**nr**	/
4	**0.8**	**0.6**	0.2	+	+	17.3	17.2	0.1	**9.6**	**9.4**	0.2	2.9	2.5	0.4	18.5	19.8	1.3	12.5	12.6	0.1
5	8.0	7.0	1.0	-	-	17.4	16.2	1.2	5.4	6.0	0.6	7.0	6.0	1.0	20.0	19.5	0.5	11.6	11.6	0.0
6	3.8	3.5	0.3	-	-	18.3	18.5	0.2	6.1	6.4	0.3	3.0	4.0	1.0	24.9	25.2	0.3	14.7	14.3	0.4
7	**nr**	**nr**	/	/	/	**nr**	**nr**	/	**nr**	**nr**	/	**nr**	**nr**	/	**nr**	**nr**	/	**nr**	**nr**	/
8	**nr**	**nr**	/	/	/	**nr**	**nr**	/	**nr**	**nr**	/	**nr**	**nr**	/	**nr**	**nr**	/	**nr**	**nr**	/

MPS = mucopolysaccharidosis; *N* = patient number; MEPs = motor evoked potentials; R = right; L = left; ID = interside difference; - = absent; + = present; amp = amplitude; CMCT = central motor conduction time; nr = value not recordable due to the absence of the transcranially-induced motor response (MEP latency column) or the absence of the response by cervical or lumbar nerve root stimulation (CMCT column); numbers in italics = reference values [32]; numbers in bold = pathological values.
